# [Corrigendum] DNA methylation is involved in the aberrant expression of miR‑133b in colorectal cancer cells

**DOI:** 10.3892/ol.2023.13998

**Published:** 2023-08-07

**Authors:** Lv Lv, Jianyu Zhou, Changwei Lin, Gui Hu, Lu Yi, Juan Du, Kai Gao, Xiaorong Li

Oncol Lett 10: 907–912, 2015; DOI: 10.3892/ol.2015.3336

Subsequently to the publication of the above paper, an interested reader drew to the authors’ attention that three of the panels shown in the upper row of Fig. 4 on p. 910, portraying the results of Transwell invasion assay experiments, showed some overlapping sections, such that the data appeared to have been derived from the same original source, where they were intended to show the results of differently performed experiments.

The authors have re-examined their data and realized that Fig. 4 was assembled incorrectly; however, the authors were able to reassemble this Figure based on the results they obtained from one of their repeated experiments, and the revised version of Fig. 4 is shown below. The authors regret the errors that were made during the preparation of the Figure, although they were able to confirm that these errors did not seriously affect the conclusions reported in the paper. The authors are grateful to the editor of *Oncology Letters* for allowing them the opportunity to publish a Corrigendum, and all the authors agree to this Corrigendum. Furthermore, they apologize to the readership for any inconvenience caused.

## Figures and Tables

**Figure 4.**
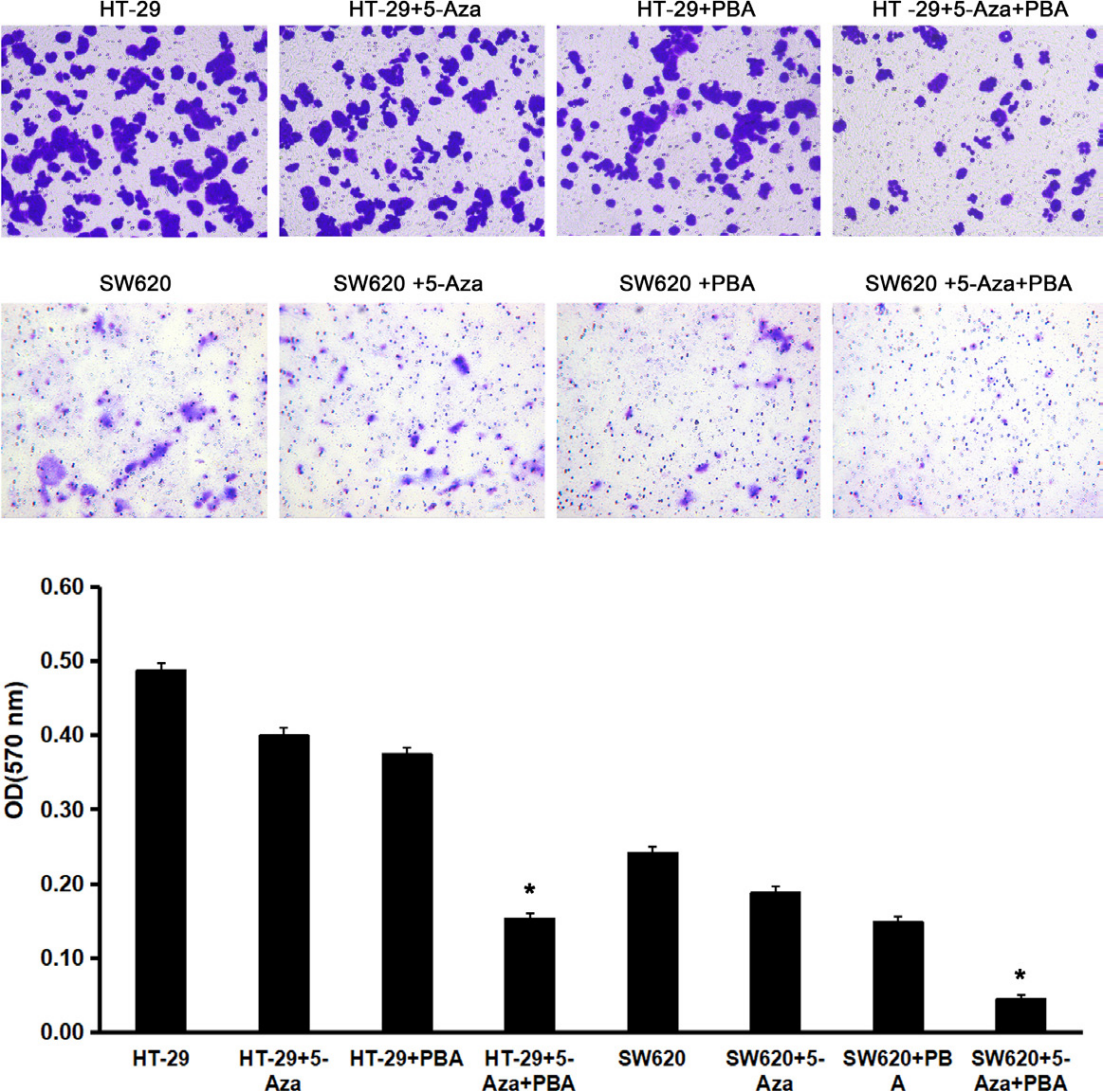
miR-133b demethylation inhibits tumor cell invasion. Colorectal cancer HT-29 and SW620 cells were treated with 5-Aza, PBA and 5-Aza/PBA. The number of invading tumor cells was calculated by analyzing Transwell invasion assays, and the miR-133b demethylation and control groups were compared. *P<0.05 vs. untreated control cells. miR, microRNA; 5-Aza, 5-aza-2’-deoxycytidine; PBA, 4-phenylbutyric acid; OD, optical density.

